# Geographic variation and factors associated with rates of knee arthroplasty in Korea-a population based ecological study

**DOI:** 10.1186/s12891-019-2766-y

**Published:** 2019-09-02

**Authors:** Agnus M. Kim, Sungchan Kang, Jong Heon Park, Tae Ho Yoon, Yoon Kim

**Affiliations:** 1 Department of Health Policy and Management, Seoul National University College of Medicine, 103 Daehak-ro, Jongno-gu, Seoul, Republic of Korea; 2 Graduate School of Public Health, Seoul National University, Seoul, Republic of Korea; 3 National Health Insurance Service, Wonju, Republic of Korea; 4 Department of Preventive & Occupational Medicine, School of Medicine, Pusan National University, Pusan, Republic of Korea; 5 Institute of Health Policy and Management, Medical Research Center, Seoul National University, Seoul, Republic of Korea

**Keywords:** Knee arthroplasty, Knee replacement, Republic of Korea, Beds, Utilization

## Abstract

**Background:**

The recent increase in knee arthroplasty (KA) use in Korea is among the highest in the world. The rapid increase in KA use suggests that the KA use in Korea could have been affected by medically unjustifiable factors. This study aimed to examine the geographic variation in the rate of KA and its associated factors in Korea.

**Methods:**

We used the data from the National Health Insurance in Korea in 2013, from which a total of 67,086 claims for KA were obtained. We calculated the age-sex-standardized KA rates of the entire population and the crude rates of the age groups 0–64 and 65 and over in 251 districts. We assessed the geographic variation of the KA rates and examined the associated factors with a multivariate linear regression with the KA rate as a dependent variable.

**Results:**

The overall rate of KA in Korea was 132.7 per 100,000 persons. The rates of KA showed a four-fold variation. The deprivation index score and the number of beds in the small to medium sized hospitals showed a positive association with the rates of KA while the number of orthopedic surgeons showed a negative association.

**Conclusions:**

Korea has been experiencing a rapid increase in the use of KA for the last decade or so, which was most prominent among the elderly population aged 65 and older. Our results suggest that the higher rate of KA is strongly related to a higher supply of beds and the socioeconomically deprived conditions. Considering that the decision concerning KA has room for discretion and also affects a considerable portion of health care expenditures, the use of KA should be thoroughly monitored with more emphasis on standardization in the decision making process and preventive measures that can lessen the need for KA.

**Electronic supplementary material:**

The online version of this article (10.1186/s12891-019-2766-y) contains supplementary material, which is available to authorized users.

## Background

The increasing incidence of knee arthroplasty (KA) is a universal phenomenon among developed countries [1–4]. KA is the most frequently performed surgical procedure in the US besides maternal and neonatal ones [5, 6]. The rate of KA increased by 62% during 2002–2015 in the US [5] and is expected to rise further [7]. Likewise, most developed countries had a rapid and continual growth in the use of KA [1, 8]. The increasing utilization of KA can be considered an inevitable consequence of the recent demographic changes and widespread acceptance of its benefits [8].

However, despite the prevalent trend of increase, there is a large variation in the rates of KA within and among countries [1, 8–11]. This variation raises a question regarding the optimal use of KA. This question is important given the escalating burden of osteoarthritis and obesity as well as population aging, as KA will affect an increasingly large population and health care expenditures. Investigating geographic variation in the KA rates and its factors can provide a clue regarding the justifiability of KA use, as variation studies can elucidate the influence of medically unjustifiable factors.

This issue is especially relevant in Korea. KA is the 6th most common surgical procedure in Korea, and, in terms of overall cost, it is the second highest [12]. The recent increase in KA use in Korea is among the highest in the world [8]. The rate of KA in Korea increased by 122% during 2006–2016 [12]. This is far higher than the increase in the population aged 65 and over of 52% [13] and in the prevalence of obesity [14] and arthritis whose increases were about 7% each during the same period [15]. The rapid increase in KA suggests that KA use in Korea could have been affected by factors which are not directly related to medical conditions. This is highly probable in view of the health care system in Korea. Korea provides universal health care through the National Health Insurance System, and the payment system is largely based on the fee-for-service structure [16]. These characteristics can lead to overuse of health care services both on the patients’ and providers’ side in that they lessen the financial burden on the patients and can motivate the providers to provide more service. Analyzing the geographic variation in the KA utilization rate in Korea would identify the factors for the overuse of KA. This study aimed to examine the geographic variation in the rate of KA and its associated factors. We first assessed the degree of variation as it suggests the likelihood that it was affected by factors which are not clinical, and using a regression model, we investigated how demographic and supply related factors were associated with the rates of KA.

## Materials and methods

### Study population and data

We used the claims database of the 2013 period from the National Health Insurance (NHI) Service, which covers the entire Korean population. The claims with the procedural code for primary total KA (N2072), primary unicompartmental KA (N2712), revision total KA (N3712, N3722), and revision unicompartmental KA (N4712, N4711) were identified. The data were provided by the NHI Service in 2015 and were previously used for the studies of geographic variation in medical services utilization in Korea [17, 18].

### Utilization rate of KA

Utilization rates of KA were calculated for 251 districts in Korea based on the patient’s place of residence. The rate was defined as the number of incidences per 100,000 persons. For the entire population of all ages, the age- and sex-standardized rates were calculated, and the Korean resident population of 2013 was used for the standardization [19]. For the cluster rates of younger than 65 (0–64), and 65 and older, crude rates were calculated.

### Description of geographic variation

To estimate the geographic variation in the rates of KA, we calculated the ratio of the rate in the area in the 90th to the 10th percentile of the distribution (P90/P10), the coefficient of variation (CV), and the systematic component of variation (SCV). The CV is the ratio of the standard deviation to the mean [17]. The SCV is a metric for measuring the true part of variation due to variation across areas by removing the random part of variation due to within-region variation [20–22]. SCV is calculated on the basis of the difference between the observed and expected value. The SCV was presented as being multiplied by 100, as suggested by Mcpherson et al. [17, 21].

### Geographic units

We applied two areal levels to the explanatory variables according to their characteristics. For variables concerning demographics and primary care, a district was applied as a geographic unit. The district (referred to as Si/Gun/Gu in Korea) is the basic administrative unit in Korea. With the average of 204,733 and median of 168,461, the range of the population number of the district goes from 10,524 to 668,415. However, for the hospital-level service, applying the district level can be misleading because those services correspond to broader utilization areas. Therefore, concerning hospital service and orthopedic care, we applied a hospital service area (HSA) [23]. The HSA is a combined areal unit composed of districts, which were constructed by hospital utilization patterns of the residents of the districts. Three criteria were applied to organize the hospital service area: 1. Minimum score of localization index - the proportion of health care use by the residents in an area [18]: 40%, 2. Minimum population size: 150,000, and 3. Maximum transportation time within a region by car: 60 min. We used the HSA based on data for the acute hospitalizations in Korea which occurred during 2011 and 2015 [24].

### Analysis of factors for geographic variation

We primarily performed a correlation analysis to investigate the relationships among independent variables and the KA rates and performed a multivariate linear regression with the rates of KA as outcome variables.

We used three explanatory variables based on the district level. First, to indicate the socio-economic conditions of the residents of a region, we used 1) the deprivation index. It is a composite measure indicating socio-economic deprivation of a region [25]. The other district-level explanatory variables are 2) the spending for musculoskeletal diseases per capita, and 3) the number of primary care physicians (per 100,000 persons). The spending for musculoskeletal diseases per capita is the sum of the out-of-pocket expense and national health insurance coverage. It was added to adjust the effect of the health care cost in assessing the association between the deprivation score and KA rate. We calculated the spending per capita by dividing the total amount of spending by the number of people in each district. The number of primary care physicians was included as a variable as the conservative treatment of knee arthritis, which would delay the KA, is commonly performed by primary care providers in Korea [26, 27]. As there is no institutionally defined group of primary care physicians in Korea [16], we used an operational definition of primary care provider based on the practice characteristics. The number of primary care physicians was calculated based on the criteria suggested by Lee et al., which defined primary care physicians by the characteristics of patients who visited the clinics [28]. They defined the primary care physicians as physicians in the clinics where the proportion of the visits with 52 simple and minor disease groups (SMDGs) [26] was over the average (38.3%) of total clinics [29]. SMDG is the diseases, suggested by the Ministry of Health and Welfare in Korea, which are considered appropriate to be treated at the primary care level. These last two explanatory variables were obtained from the Health Care Resources & Service Information Report issued by the Ministry of Health and Welfare [30].

The HSA-level explanatory variables are 4) the number of orthopedic surgeons (per 100,000 persons), 5) the number of beds in hospitals with less than 300 beds for small to medium sized hospitals (per 1000 persons), and 6) the number of beds in hospitals with more than 300 beds for large sized hospitals (per 1000 persons) [18]. The number of orthopedic surgeons consists of the active orthopedic surgeons working in the clinics and hospitals except for the long-term care hospitals. The data were provided by the Health Care Resources & Service Information Report issued by the Ministry of Health and Welfare [30]. All the analyses were conducted using SAS, version 9.3 (SAS Institute, Inc., Cary, NC, USA) and SPSS 23 (IBM Corporation, Armonk, NY, USA).

## Results

**Fig. 1:**
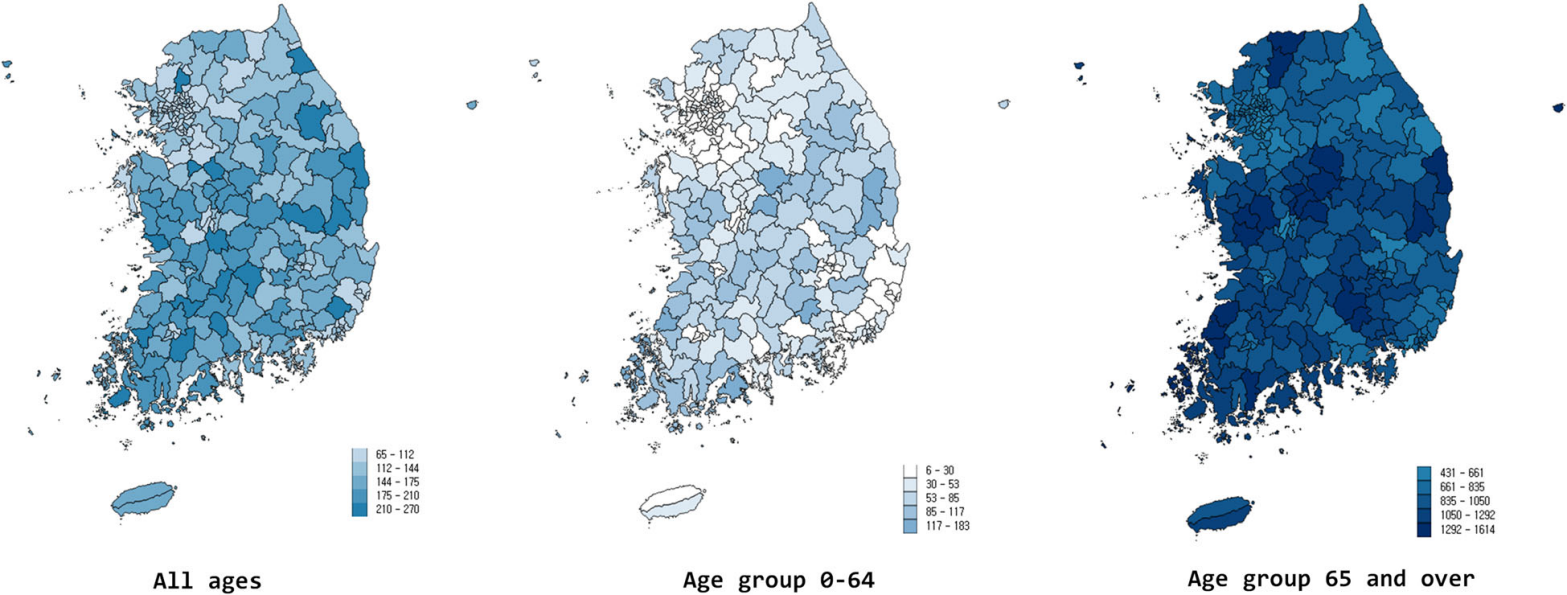
This geographic distribution of rates of knee arthroplasty per 100,000 in Korea in 2013

There were a total of 67,086 KA in Korea in 2013. The national rate of KA was 132.7 per 100,000 persons (Table 1). Except for the persons aged 40 and under, women showed a higher rate of KA, and the rate of the female was about 6 times that of the male. Age specific national rates were 31.7 and 878.2 for age groups 0–64 and 65 and over respectively, and the highest rate was 1066.4 for ages 70–74. Figure 1 presents maps of KA rates. The variation statistics of KA rates are presented in Table 2. Rates in the highest districts were more than four times higher than in the lowest district, and the rate gap was the largest in age group 0–64 with a 29-fold difference. Concerning the CV, the measure of dispersion insensitive to the scale, it was the most prominent in the age group 0–64 at 0.7 while the CV in the rates for all ages and the age group 65 and over were both 0.3.

**Table 1:** Absolute frequencies and rates of knee arthroplasty per 100,000 persons by age group

Age group	Absolute frequencies of knee arthroplasty (Rates per 100,000)		
	Male	Female	Total
0–40	82 (0.5)	55 (0.4)	137 (0.5)
45–49	58 (2.7)	198 (9.6)	256 (6.1)
50–54	243 (11.1)	1097 (51.1)	1340 (30.9)
55–59	621 (36.1)	3362 (194.5)	3983 (115.5)
60–64	1128 (95.0)	7282 (589.0)	8410 (347.1)
65–69	1994 (219.6)	12,751 (1259.7)	14,745 (767.9)
70–74	2385 (306.1)	16,515 (1662.7)	18,900 (1066.4)
75 +	2633 (326.3)	16,682 (1089.6)	19,315 (826.1)
Overall	1047.0 (36.2)	810.1 (229.2)	882.0 (132.7)

The denominator is the number of people in Korea of the corresponding age and sex group in the Korean Resident Population in 2013 provided by the Statistics Korea

**Table 2:** Variation statistics of the rates of knee arthroplasty

	National average rate	Max	Min	P90/P10	CV	SCV
The rates of KA (standardized- all ages)	132.7	270.5	64.6	2.1	0.3	8.5
The rates of KA (0–64)	31.7	183.3	6.3	5.2	0.7	26.1
The rates of KA (65 and over)	878.2	1614.5	430.9	2.0	0.3	7.2

*CV* coefficient of variation (the ratio of standard deviation to the mean), *SCV* systematic component of variation

The correlation analysis among the variables is presented in Table 3. All the explanatory variables, except the number of large sized hospitals, showed significant correlations with the rates of KA. Deprivation index score had positive correlations with the rates of KA, which suggests that the more deprived an area is, the higher the rate of KA. The positive correlation between the number of beds in small to medium sized hospitals and the rates of KA suggests more supply of beds in small to medium sized hospitals is associated with higher rates of KA. The number of orthopedic surgeons and the number of primary care physicians had negative correlations with the KA rates, which suggests that a higher number of either of them is associated with lower KA rates.

**Table 3:** Correlation matrix of dependent and independent variables

	Rate (all)	Rate (0–64)	Rate (65 +)	Dep.	Mus. Cost	PCP	OS	Beds (Small)	Beds (Large)
Rate (all)	1								
Rate (0–64)	.814**	1							
Rate (65 +)	.964**	.687**	1						
Dep.	.697**	.839**	.626**	1					
Mus. Cost	−.221**	−.245**	−.198**	−.182**	1				
PCP	−.428**	−.468**	−.435**	−.440**	.659**	1			
OS	−.301**	−.229**	−.303**	−.149*	.290**	.399**	1		
Beds (Small)	.385**	.373**	.392**	.478**	.105	−.224**	−.047	1	
Beds (Large)	−.106	−.044	−.115	−.071	.174**	.252**	.542**	−.171**	1

**p < 0.01, Dep, deprivation index score; Mus. Cost, Average costs of musculoskeletal diseases; PCP, the number of primary care physicians; OS, the number of orthopedic surgeons; B (< 300), the number of beds in small to medium sized hospitals; B (> 300), the number of beds in large sized hospitals

According to the regression analysis (Table 4), the deprivation index score was positively associated with the rates of KA, which means the higher deprivation in an area was associated with the higher rate of KA. A one unit increase in the deprivation index score was associated with a 6.5 unit increase in the KA rate. Considering that the deprivation score ranges from − 10.5 to 7.2 with the mean at 0.0 (Additional file 1: Table S1), the increase in the deprivation index score from the lowest to the mean is related to a 68.3 unit increase in the KA rate, which is about 51.7% of the average KA rate. Given the average rate of each age group, the impact of the deprivation index score on the KA rate is the most marked in the age group 0–64. The increase in the deprivation index score from the lowest to the mean is related to the 228.0% increase in the average KA rate, while the increase in KA rate is 40.2% in the elderly population.

**Table 4:** Regression analysis of hospitalization rates for knee arthroplasty

	All ages		Age group 0–64		Age group 65 and over	
	Coefficient	SE	Coefficient	SE	Coefficient	SE
Baseline (intercept)	183.422	11.432	66.750	6.453	1149.261	76.014
District-level						
Deprivation index	6.537***	.624	6.883***	.352	33.652***	4.151
Average costs of musculoskeletal diseases	−.021	.082	−.037	.046	.119	.547
No. of primary care physicians per 100,000	−.322	.190	−.134	.107	−2.364	1.265
Hospital service area-level						
No. of orthopedic surgeons per 100,000	−5.187***	1.319	−1.915*	.745	−32.196***	8.772
No. of small to medium sized hospital beds per 1000	2.750*	1.390	.038	.784	22.457*	9.240
No. of large sized hospital beds per 1000	4.229	2.570	2.549	1.451	27.211	17.092
Adjusted R^2^	.546		.737		.464	
F-statistics	48.489***		113.216***		34.926***	

*p < 0.05 ***p < 0.001

The number of orthopedic surgeons showed a negative association with the rates of KA. A one unit increase in the number of orthopedic surgeons per 100,000 was associated with a 5.2 unit decrease in the KA rate. Given the distribution of the number of orthopedic surgeons, the increase in the number of orthopedic surgeons from the lowest to the mean is related to a 29.0 unit decrease in the KA rate, which is about 21.9% of the average KA rate. As in the case with the deprivation index score, the impact of the number of orthopedic surgeons on the KA rate was higher in the non-elderly population than in the elderly population. The number of orthopedic surgeons from the lowest to the mean is related to the 33.8% decrease in the average KA rate in the age group 0–64 and to the 20.5% decrease in the average KA rate in the elderly population.

The number of beds in small to medium sized hospitals had a positive association with the rates of KA. Its increase from the lowest to the mean is related to a 9.9 unit increase in the KA rate, which is about 7.5% of the average KA rate. While its increase from the lowest to the mean in the age group 65 and over is about 9.2% of the average KA rate in that age group, no statistically significant association appeared in the age group 0–64. The average costs of musculoskeletal disease and the number of primary care physicians, which had a significant association with the rates of KA in the correlation analysis, did not have significant associations.

## Discussion

This study investigated geographic variation in the rate of KA in Korea and the related factors using the 2013 National Health Insurance Database in Korea. The overall rate of KA in Korea was 132.7 per 100,000 persons, and the female had a six-fold higher rate than the male. The rate of KA in the age group 65 and over was 878.2, which is about 28 times that in the age group 0–64. The rates of KA showed a high geographic variation, which was the largest in the age group 0–64 with a 28-fold difference. The deprivation index score and the number of beds in the small to medium sized hospitals showed a positive association with the rates of KA while the number of orthopedic surgeons showed a negative association.

Until the last few years, the overall rate of KA in Korea was not in a high bracket among the developed countries. However, in the case of the elderly population, the KA rate in Korea can be considered markedly high [8]. Considering the sharp increase in the KA rates in Korea during recent years, which is salient in comparison with other countries [8, 12], the overall rate is also expected to belong to the higher side. The variation in the rates of KA is known to be higher compared with other procedures, and this high variation is also shown in this study with a four-fold difference between the highest and the lowest. The high variability of surgery rates among the regions is referred to as “surgical signature,” which explains the geographic variation as a result of regional differences in physicians’ practice styles and the degree of the incorporation of patients’ opinions into the decision making rather than the patients’ characteristics and health care supply [31]. However, our study results show that the regional socioeconomic characteristics and supply of beds and surgeons do play an important role in determining the surgery rate.

The positive association between the deprivation index score and the rates of KA suggests that the residents in more deprived areas are more likely to receive knee arthroplasty. The relationship between these two variables has been mixed. While the positive association between socioeconomic deprivation and KA rates has been reported [1, 11, 32], there were studies showing lower KA use in persons with lower incomes [33, 34]. The latter relationship can be explained with limited access to care among the deprived population in terms of geographic and financial factors. However, in a health care system where a considerable part of the expense for medical procedures is covered by insurance, the significance of financial access to care in explaining geographic variation in health care use is reduced.

Therefore, the positive association between the socioeconomic deprivation and the rates of KA can be explained in two ways. First, it is likely to reflect the higher possibility that the persons in deprived areas have conditions requiring KA [35]. Higher prevalence of obesity and arthritis among persons with lower incomes suggests that a greater proportion of persons from deprived areas are susceptible to those conditions. In Korea, the higher prevalence of obesity and arthritis in persons with lower incomes and persons from disadvantaged areas has been documented [36, 37]. Second, the people in the deprived areas are less likely to receive the proper treatments that can prevent the occurrence or progress of knee arthritis. This can be related to their living and working environments as well as to the provision of medically preventive measures. The relationship between the socioeconomic deprivation and high KA rate should be further investigated considering these possibilities.

The number of orthopedic surgeons showed a negative association with the rates of KA. The relationship of the two was indefinite in prior studies. While the negative relationship has been documented in a study from Germany [1], no association between the two variables has been reported in other studies [9, 32, 38]. The negative association of the number of orthopedic surgeons with the rates of KA in Korea can be explained in two ways. First, better access to orthopedic care could have delayed the surgery with more preventive and conservative measures. Second, the number of the orthopedic surgeons in this study includes the orthopedic surgeons who are committed to outpatient care in clinics as well as orthopedic surgeons in hospitals. For the outpatient-oriented surgeons, maintaining outpatient care can be a more attractive option than recommending a KA. These hypotheses can explain the negative impact of the number of orthopedic surgeons on KA rates. But to accurately measure the impact of the orthopedic surgeons on KA rates, the number of orthopedic surgeons needs to be divided according to their practice patterns.

The positive association between the number of hospital beds and the rate of KA suggests the possibility that the supply of beds could have induced the use of KA. Although the safety and benefits of KA are widely recognized, the conditions for KA are not life-threatening and do not require immediate surgery. Therefore, the patient’s decision would be dependent on the opinion of the orthopedic surgeon who takes care of the patient. The positive relationship between the number of beds in small to medium sized hospitals and the rate of KA suggests that hospital based orthopedic surgeons are more likely to recommend KA and that this tendency is prominent in small to medium sized hospitals in Korea. This is probable given the recent increase in the number of small to medium sized hospitals and competition among them.

What is noticeable is the difference in the influence of the explanatory variables on the rate of KA among the age groups 0–64 and 65 and over. Given the average KA rate of each age group, the relative impact of the deprivation index score and the number of orthopedic surgeons was greater in the age group 0–64; however, the influence of the small to medium sized hospital beds did not appear in the age group 0–64. These results show that KA use induced by supply of beds is more likely to happen in the aged population.

There are several limitations in this study. First, in terms of diagnostic criteria, the subjects in this study include both the primary and revision procedures and the analysis was not performed separately. Given the small percentage of the revision of about 4% as of 2017 [41], this would not have affected the estimation of the association between the independent variables and the KA rates. However, considering the increasing use of revision and the different characteristics concerning indication, a separate analysis would be useful to clarify the distinctive traits between the two procedures. Second, a study based on a regional unit risks the ecological fallacy. However, the two main factors in this study, regional socioeconomic conditions and supplier factors, have their own significance in the regional unit analysis as distinct from that based on the individual unit. Third, the data used in this study were not specified in terms of the patient conditions. The data classified by the cause of KA would have made the interpretation and policy implication of our study clearer. Fourth, the practice pattern of orthopedic surgeons in Korea differs according to the facilities where they practice. Unlike the orthopedic surgeons in the hospitals, those in the clinics focus mainly on outpatient care. However, the proportion of orthopedic surgeons who do not perform surgery and the gap in the surgery volume among the orthopedic surgeons have not been considered in this study. As the surgery volume of each orthopedic surgeon is an important factor for the use of KA from the perspective of patients, the impact of surgery volume should be further investigated. Lastly, this study is a cross-sectional one, which involves a limitation in exploring the causative relationship between the explanatory variables and the rate of KA. For a more accurate assessment of the influence of the factors revealed in our study, an analysis based on the longitudinal data should be performed.

## Conclusions

Korea has been experiencing a rapid increase in the use of KA for the last decade. Our results show that the socioeconomic deprivation and the supply of beds in small to medium sized hospitals were associated with the higher rates of KA and the supply of orthopedic surgeons with the lower rates of KA. The influence of the socioeconomic deprivation on the KA rates needs to be further investigated in connection with the general working and life circumstances as well as the provision of proper preventive measures. The prominent impact of supply of hospital beds on the KA rate among the elderly also needs to be further examined as this relationship suggests a medically unjustifiable inducement. Considering that the decision concerning the KA has room for discretion and also affects a considerable portion of health care expenditures, the use of KA should be thoroughly monitored with more emphasis on standardization in the decision making process and preventive measures that can lessen the need for KA.

## Additional file

Additional file 1: **Table S1.** Characteristics of the independent variables (DOCX 13 kb)

## Data Availability

The data are available from the first author.

## References

[1] Schafer T, Pritzkuleit R, Jeszenszky C, Malzahn J, Maier W, Gunther KP, Niethard F (2013). Trends and geographical variation of primary hip and knee joint replacement in Germany. Osteoarthritis Cartilage.

[2] Robertsson O, Dunbar MJ, Knutson K, Lidgren L (2000). Past incidence and future demand for knee arthroplasty in Sweden: a report from the Swedish knee arthroplasty register regarding the effect of past and future population changes on the number of arthroplasties performed. Acta Orthop Scand.

[3] Culliford D, Maskell J, Judge A, Cooper C, Prieto-Alhambra D, Arden NK (2015). Future projections of total hip and knee arthroplasty in the UK: results from the UK clinical practice research datalink. Osteoarthr Cartil.

[4] de Pina MF, Ribeiro AI, Santos C. Epidemiology and variability of orthopaedic procedures worldwide. In: European Instructional Lectures. Berlin: Springer; 2011. p. 9–19.

[5] Fingar K, Stocks C, Weiss A, Steiner C (2014). Most frequent operating room procedures performed in US hospitals, 2003–2012. HCUP statistical brief# 186.

[6] HCUP Fast Stats. Healthcare Cost and Utilization Project (HCUP). March 2019. Agency for Healthcare Research and Quality, Rockville, MD. www.hcup-usus.hrq.gov/faststats/national/inpatientcommonprocedures.jsp?year1=2015&characteristic1=0&included1=1&year2=&characteristic2=0&included2=1&expansionInfoState=hide&dataTablesState=hide&definitionsState=hide&exportState=hide.

[7] Inacio MCS, Paxton EW, Graves SE, Namba RS, Nemes S (2017). Projected increase in total knee arthroplasty in the United States - an alternative projection model. Osteoarthritis Cartilage.

[8] Pabinger C, Lothaller H, Geissler A (2015). Utilization rates of knee-arthroplasty in OECD countries. Osteoarthr Cartil.

[9] Judge A, Welton NJ, Sandhu J, Ben-Shlomo Y (2009). Geographical variation in the provision of elective primary hip and knee replacement: the role of socio-demographic, hospital and distance variables. J Public Health.

[10] Fisher ES, Tomek IM, Esty AR, Goodman DC, Bronner KK (2010). Trends and regional variation in hip, knee, and shoulder replacement.

[11] Dixon T, Urquhart DM, Berry P, Bhatia K, Wang Y, Graves S, Cicuttini FM (2011). Variation in rates of hip and knee joint replacement in Australia based on socio-economic status, geographical locality, birthplace and indigenous status. ANZ J Surg.

[12] Statistics Korea: Number and costs of surgical procedures in Korea 2006-2018; 2019. http://kosis.kr/statHtml/statHtml.do?orgId=350&tblId=TX_35004_A003&conn_path=I3. Accessed 20 June 2019.

[13] Statistics Korea: Korean Resident Population; 2019. http://kosis.kr/statHtml/statHtml.do?orgId=101&tblId=DT_1B040M5. Accessed 20 June 2019.

[14] Statistics Korea: Trends in obesity prevalence among adults in Korea 2005–2016; 2018. http://kosis.kr/statHtml/statHtml.do?orgId=117&tblId=DT_11702_N101#. Accessed 20 June 2019.

[15] Ministry of Health & Welfare (2018). The proportion of persons (aged 50 and over) diagnosed with arthritis 2008-2017.

[16] Kim AM, Park JH, Yoon TH, Kim Y (2019). Hospitalizations for ambulatory care sensitive conditions as an indicator of access to primary care and excess of bed supply. BMC Health Serv Res.

[17] Kim AM, Park JH, Kang S, Hwang K, Lee T, Kim Y (2016). The effect of geographic units of analysis on measuring geographic variation in medical services utilization. J Prev Med Public Health.

[18] Kim AM, Park JH, Kang S, Kim Y (2017). Evaluation of geographic indices describing health care utilization. J Prev Med Public Health.

[19] Statistics Korea. Resident Population in five-Year Groups 2013; 2016. http://kosis.kr/statisticsList/statisticsListIndex.do?menuId=M_01_01&vwcd=MT_ZTITLE&parmTabId=M_01_01?menuId=M_01_01&vwcd=MT_ZTITLE&parmTabId=M_01_01&parentId=A. Accessed 20 June 2019.

[20] Kim AM. Effect of regional characteristics on regional variation in medical services utilization. thesis. Seoul: Seoul National University; 2016. (Korean)

[21] McPherson Klim, Wennberg John E., Hovind Ole B., Clifford Peter (1982). Small-Area Variations in the Use of Common Surgical Procedures: An International Comparison of New England, England, and Norway. New England Journal of Medicine.

[22] Appleby J, Raleigh V, Frosini F, Bevan G, HaiYan G, Lyscom T, Gao H (2011). Variations in health care: the good, the bad and the inexplicable.

[23] Kim AM, Park JH, Kang S, Yoon TH, Kim Y (2019). An ecological study of geographic variation and factors associated with cesarean section rates in South Korea. BMC Pregnancy Childbirth.

[24] Kim Y, Lee TS, Lee HY, Hwang KS, Lee JY, et al. NHIS health map project; 2016. (Korean)

[25] Townsend P (1987). Deprivation. J Soc Policy.

[26] Kim Agnus, Cho Seongcheol, Kim Hyun, Jung Hyemin, Jo Min-Woo, Lee Jin, Eun Sang (2018). Primary Care Patients’ Preference for Hospitals over Clinics in Korea. International Journal of Environmental Research and Public Health.

[27] Kim AM, Kang S, Park JH, Yoon TH, Kim Y (2019). A spatial analysis of geographic variation and factors associated with hospitalization for bacterial pneumonia in Korea. BMC Pulm Med.

[28] Lee JY, Eun SJ, Kim HJ, Jo M-W (2016). Finding the primary care providers in the specialist-dominant primary care setting of Korea: a cluster analysis. PLoS One.

[29] Park SK, Kim JE, Lee HJ, Cho SJ, Han SJ. National Health and Medical Service Statistics. Sejong: Ministry of Health & Welfare; 2018.

[30] Park S, Kim J, Cho S, Lee H, Han S, et al. Health Care Resources & Service Information Report. Sejong: Korea Institute for Health and Social Affairs Ministry of Health and Welfare; 2017. (Korean)

[31] Birkmeyer JD, Reames BN, McCulloch P, Carr AJ, Campbell WB, Wennberg JE (2013). Understanding of regional variation in the use of surgery. Lancet.

[32] Weinstein JN, Bronner KK, Morgan TS, Wennberg JE (2004). Trends and geographic variations in major surgery for degenerative diseases of the hip, knee, and spine. Health Affairs (Project Hope).

[33] Dixon T, Shaw M, Ebrahim S, Dieppe P: Trends in hip and knee joint replacement: socioeconomic inequalities and projections of need. Ann Rheum Dis 2004, 63. 10.1136/ard.2003.012724 PMC1755069 15194578

[34] Mahomed NN, Barrett J, Katz JN, Baron JA, Wright J, Losina E (2005). Epidemiology of total knee replacement in the United States Medicare population. JBJS.

[35] Hawker GA, Wright JG, Glazier RH, Coyte PC, Harvey B, Williams JI, Badley EM (2002). The effect of education and income on need and willingness to undergo total joint arthroplasty. Arthritis Rheum.

[36] Control CD, Prevention KY, Oh K (2015). Prevalence of chronic diseases by household income among adults in Korea, 2013. Public Health Wkly Rep.

[37] Cho Y. Being male, elderly, and poor makes one more vulnerable to obesity. In: Sisa Journal. Seoul; 2016. http://www.sisapress.com/journal/article/158718. Accessed June 20 2019. (Korean)

[38] Coyte PC, Hawker G, Wright JG. Variations in knee replacement utilization rates and the supply of health professionals in Ontario, Canada. J Rheumatol. 1996;23(7):1214-20. 8823695

