# Supplementary material for: Geographic variation and factors associated with rates of knee arthroplasty in Korea-a population based ecological study
Source: BMC Musculoskelet Disord. 2019 Sep 2;20:400. doi: 10.1186/s12891-019-2766-y (PMC6721190; doi:10.1186/s12891-019-2766-y)
Supplement: Supplementary file 1 — Table S1. Characteristics of the independent variables (DOCX 13 kb) [file 12891_2019_2766_MOESM1_ESM.docx]

Additional Table 1. Characteristics of the independent variables

| Variables | Mean | Minimum | Maximum | Standard deviation |
| --- | --- | --- | --- | --- |
| District-level |  |  |  |  |
| Deprivation index | 0.0 | -10.5 | 7.2 | 3.6 |
| Average costs of musculoskeletal diseases | 26.4 | 0.0 | 292.0 | 31.0 |
| No. of primary care physicians per 100,000 | 29.8 | 4.8 | 140.3 | 14.9 |
| Hospital service area-level |  |  |  |  |
| No. of orthopedic surgeons per 100,000 | 9.4 | 3.8 | 12.1 | 1.8 |
| No. of small to medium sized hospital beds per 1,000 | 3.6 | 0.0 | 6.8 | 1.6 |
| No. of large sized hospital beds per 1,000 | 1.7 | 0.0 | 4.0 | 0.9 |
